# Supplementary material for: Resolving the full spectrum of human genome variation using Linked-Reads
Source: Genome Res. 2019 Apr;29(4):635–45. doi: 10.1101/gr.234443.118 (PMC6442396; doi:10.1101/gr.234443.118)
Supplement: Supplemental Material [file supp_gr.234443.118_Supplemental_longranger-2.2.zip › longranger-2.2.tar.bz2!/lr2.2-public/lib/stan/cmdstan/stan/doc/api/html/rev_2functions_2log10_8hpp_source.html]

Stan: src/stan/agrad/rev/functions/log10.hpp Source File


|  |  |
| --- | --- |
|  | Stan  2.5.0  probability, sampling & optimization |


- Main Page
- Related Pages
- Namespaces
- Classes
- Files

- File List
- File Members

All Classes Namespaces Files Functions Variables Typedefs Enumerations Enumerator Friends Macros Pages

- src
- stan
- agrad
- rev
- functions

log10.hpp

Go to the documentation of this file.

1 #ifndef STAN\_\_AGRAD\_\_REV\_\_FUNCTIONS\_\_LOG10\_HPP

2 #define STAN\_\_AGRAD\_\_REV\_\_FUNCTIONS\_\_LOG10\_HPP

3

4 #include <cmath>

5 #include <stan/agrad/rev/var.hpp>

6 #include <stan/agrad/rev/internal/v\_vari.hpp>

7 #include <stan/math/constants.hpp>

8

9 namespace stan {

10  namespace agrad {

11

12  namespace {

13  class log10\_vari : public op\_v\_vari {

14  public:

15  const double exp\_val\_;

16  log10\_vari(vari\* avi) :

17  op\_v\_vari(std::log10(avi->val\_),avi),

18  exp\_val\_(avi->val\_) {

19  }

20  void chain() {

21  avi\_->adj\_ += adj\_ / (stan::math::LOG\_10 \* exp\_val\_);

22  }

23  };

24  }

25

55  inline var log10(const var& a) {

56  return var(new log10\_vari(a.vi\_));

57  }

58

59  }

60 }

61 #endif

stan::math::LOG\_10

const double LOG\_10

The natural logarithm of 10, .

**Definition:** constants.hpp:38

stan::agrad::var::vi\_

vari \* vi\_

Pointer to the implementation of this variable.

**Definition:** var.hpp:40

v\_vari.hpp

exp\_val\_

const double exp\_val\_

**Definition:** log10.hpp:15

var.hpp

stan::agrad::var

Independent (input) and dependent (output) variables for gradients.

**Definition:** var.hpp:27

constants.hpp

stan::agrad::log10

fvar< T > log10(const fvar< T > &x)

**Definition:** log10.hpp:15


---

[ Stan Home Page ]

*© 2011–2014,
Stan Development Team.*
